# Social inequality and children’s health in Africa: a cross sectional study

**DOI:** 10.1186/s12939-016-0372-2

**Published:** 2016-06-14

**Authors:** Tim B. Heaton, Benjamin Crookston, Hayley Pierce, Acheampong Yaw Amoateng

**Affiliations:** Department of Sociology, Brigham Young University, 2033 JFSB, Provo, UT 84602 USA; Department of Health Science, Brigham Young University, Provo, USA; North-west University, Mafikeng, South Africa

**Keywords:** Socioeconomic inequality, Children’s health, Africa

## Abstract

**Background:**

This study examines socioeconomic inequality in children’s health and factors that moderate this inequality. Socioeconomic measures include household wealth, maternal education and urban/rural area of residence. Moderating factors include reproductive behavior, access to health care, time, economic development, health expenditures and foreign aid.

**Methods:**

Data are taken from Demographic and Health Surveys conducted between 2003 and 2012 in 26 African countries.

**Results:**

Birth spacing, skilled birth attendants, economic development and greater per capita health expenditures benefit the children of disadvantaged mothers, but the wealthy benefit more from the services of a skilled birth attendant and from higher per capita expenditure on health.

**Conclusion:**

Some health behavior and policy changeswould reduce social inequality, but the wealthy benefit more than the poor from provision of health services.

## Background

Social determinants have a profound influence on health inequality and should receive due consideration when developing health policy [1, 2]. Sociology has long recognized that social inequality is a fundamental dimension of social institutions and there is growing interest in apply this insight to health related behavior and outcomes [3, 4]. Social determinants play a particularly important role in children’s health, survival, and nutritional status as a result of a child’s inherent vulnerability and reliance on others to protect their health [5]. Research has identified disparities in child mortality and nutritional status associated with socioeconomic factors in many different contexts [6, 7]. Reducing socioeconomic disparity is one important means of improving child health globally. The purpose of this study is to document social inequality in child health in Africa and identify possible factors that reduce the deleterious impact of socio-economic determinants on child nutritional status and child survival. First, the project will estimate the association between key socio-economic indicators (education, urban residence and household wealth) and standard measures of child health (infant mortality, survival through age five and nutritional status). We address this question in regression models predicting each health outcome. The second objective is to assess the degree to which critical healthy behaviors at the individual level (birth spacing, delivering with a skilled birth attendant and immunization) and macro-level factors (economic development, health expenditures, foreign aid for water development, and change over time) moderate the influence of socio-economic indicators on health outcomes. We address this issue by including interaction terms between socio-economic characteristics, healthy behaviors and macro-level factors. Children born in Africa are at great risk of undernutrition and death. More than one million (1,208,000) babies die before they reach 1 month of age [8] while another three million (3,192,000) children, who survived their first month of life, die before their first birthday [9]. Child undernutrition is more common in Africa than any other region of the world. Child stunting, a measure of chronic undernutrition, exceeds forty percent in several African countries (http://www.measuredhs.com). Even though the Millennium Development Goals (MDGs) on child health in many sub-Saharan African nations lagged far behind target, there was progress in several low-income countries. While the MDGs targeted for 2015 were attained through immediate strategic investments in selected evidence-based interventions and targeted health systems strengthening in some areas, this was not the case in most African countries [10].

Unfortunately, even though these trends show promise, they are the exception rather than the rule. With a toll of more than 13,000 deaths per day, sub-Saharan Africa accounts for more half of the world’s maternal and child deaths. In addition, an estimated 880,000 babies are stillborn in sub-Saharan Africa each year and remain invisible on the policy agenda [11]. It is against the background of the limited progress and socioeconomic inequality in health outcomes in 26 sub-Saharan Africa that we undertake the current project. We use several waves of the Demographic and Health Survey data to examine the possibility that improved healthy behavior, economic growth, and greater investment in health will contribute to better child health and lower socioeconomic disparities.

### Key social determinants

Child health has been linked to socio-economic conditions including household income, maternal education, paternal education, household size, household structure, employment, and indicators of standard of living [12–16]. In addition to the direct influence these factors exert on health, they also largely determine whether family members are able to maintain standards of cleanliness, access goods and services, and have food security [13, 15]. Economic status has been singled out as a key social determinant in the millennial development goals, but maternal education [17, 18], and type of residence [19] are also important. It is important to disentangle the relative importance of socioeconomic variables because of their policy implications [20].

### Moderating factors

The theory of fundamental causes implies that health outcomes can be improved by weakening the link between socioeconomic factors and health outcomes [2]. While recent policy discussions have addressed the impact of social determinants (http://www.who.int/social_determinants/en/), [21], several factors inhibit policy attempts to reduce socioeconomic gaps in child health [22]. “Elucidating specific mediating mechanisms is important both to understanding what the main drivers of health disparities are and to identify interventions to eliminate disparities” [23]. We consider various factors that potentially mitigate the importance of social determinants including individual behavior-- birth spacing, use of a skilled birth attendant and immunization, and macro-level factors including economic development, greater expenditure on healthcare, foreign aid to improve water, and the trend over time.

#### Birth spacing

Reproductive practices influence child nutritional status. Birth spacing is of particular importance for child health outcomes [13, 14, 17, 24]. Research demonstrates that the risk of both stunting and mortality increases with rapid childbearing [25–27]. Forste (1994) found that short preceding intervals are especially deleterious for child and maternal health. In addition, birth to conception intervals of less than 6 months are associated with increased risk of pre-term births, low birth weight and small gestational age [28]. Norton (2005) also claims that infants spaced at least 36 months apart are associated with the lowest possible mortality risk; he concludes “in 2003, if women in developing countries had no birth intervals less than 24 months, almost 2 million deaths to children under the age of five could be averted.” Stunting also substantially declines when a child is conceived more than a year and a half after the child before [24]. Research on child nutritional status also confirms the importance of prenatal and birthing care [16]. Birth spacing also mediates the relationship between education and child health [17]. To the extent that family planning can be widely distributed at relatively low cost, it is possible that women regardless of income, education or place of residence can practice healthy birth spacing. Thus, birth spacing has the potential to reduce socioeconomic inequality in child health.

#### Access to adequate health care services

Increasing access to adequate health care services is an effective step in reducing undernutrition [29]. Socioeconomic status influences children’s health through access to and utilization of health care [13, 17, 24, 30]. Further, Rutstein (2000) found an inverse relationship between child mortality and the use of medical services such as prenatal care (from a doctor or nurse), having medical attendants at birth, and giving birth in a medical facility [13]. Gage (2007) found that “while many health programs have tended to ignore contextual barriers to the use made of health services, this study found evidence for a range of area influences on the odds of utilizing maternal health services.” One such influence, particularly in rural areas, is prenatal care. Prenatal care is an important entry point into the health system that facilitates women’s access to medical care for future needs of both her and her children [31]. Kuate-defo and Diallo (2002) found that healthcare explained most of the relationship between education and mortality because education facilitated access to healthcare [12]. It follows that access to trained health professionals may reduce socioeconomic differences in child health.

Vaccinations have greatly reduced child mortality and morbidity worldwide and have led to the eradication of smallpox, and substantial reductions in poliomyelitis and measles, in addition to other diseases [32, 33]. It is estimated that expanding vaccine delivery further may result in the prevention of 6.4 million child deaths between 2011 and 2020 [34]. Vaccinations are among the most cost-effective of medical interventions and are typically a fraction of the cost of most therapeutic interventions [35, 36]. Vaccinations are a promising target for reducing child health disparities and inequities because they are inexpensive, highly effective, and easy to distribute relative to other health interventions [37].

#### Macro-level factors

Broader social trends such as time, economic development, and health care expenditures may also moderate the importance of social determinants [29]. Health systems are inequitable, providing better services to the well-off who need them less, than to the poor, who need them more. In the absence of a concerted effort to ensure that health systems reach disadvantaged groups more effectively, such inequities are likely to continue [38]. Without this concerted effort, health care expenditures continue to benefit those who are already benefiting from the care. To the degree that health policy is sensitive to the recent emphasis on health disparities, socioeconomic inequality in health outcomes should decline over time. Infant mortality rates have been declining globally and in Africa (http://www.measuredhs.com), [39]. Nutritional status has improved globally and in Sub-Saharan Africa, but the improvements in Africa have been more recent and are not evident in all countries [40]. Increasing coverage of health services has been accompanied by a decline in the economic gradient of access [41], but the economic gradient in infant mortality has increased [39]. To assess general trends, we examine the degree to which socioeconomic inequality has declined over time.

Growth in national income also has a positive impact on children’s nutritional status [42, 43], but economic growth may not be sufficient to promote substantial change if it is not accompanied by a more equitable income distribution and investments in healthcare [40]. A rising standard of living implies that individuals will have more resources to provide an adequate diet and access to basic health care. Wealthier nations will also be in a better position to enhance health services, and improve educational systems [44]. The urban/rural disparity in children’s nutritional status does decline at higher levels of development [19]. Progress in reducing child mortality in sub-Saharan Africa has been accomplished through expansion of basic health interventions including immunization, breastfeeding, supplementation and safe drinking water [39]. These interventions are relatively inexpensive and have the potential to reduce inequality in health outcomes. Equity-focused interventions that target the most marginalized population can improve overall health outcomes and reduce inequities without increasing overall cost [45]. Increasing expenditures on health care is associated with a modest decline in urban/rural differentials in health income [19].

In the case of health expenditures, Chambers and Booth (2012) found that an increase in health expenditure per capita may influence health outcomes, but research shows that this is not the whole story [46]. Focusing on African countries, the greatest health expenditure has taken place in Uganda, where outcomes have improved only a little. And outcomes remain unchanged in Niger despite a doubling in health spending between 2004 and 2009. A study by Wilson (2011) looked at development assistance for health (DAH) and found that the effectiveness has not increased over time, even as the funding has increased four-fold [47]. Without strict control, increased health expenditures and health policy may not succeed in reducing these socioeconomic disparities.

Foreign aid is often earmarked specifically for public health interventions focused on reducing child mortality and improving overall health outcomes [48]. Recent research suggests that aid has positively impacted child health in developing countries [48, 49]. Aid potentially impacts child health through improvements in healthcare systems, water and sanitation, and maternal and child nutrition. For example, improvements in clean water delivery through foreign aid are critical to the success of the Millennium Development Goals to reduce child mortality and improve maternal health and are requisite to sustaining these efforts long-term [50, 51]. Research from Bolivia indicating investments in water were correlated with declines in child mortality reinforces this view [52]. Aid has the potential to reduce health disparities to the extent that it is targeted to disadvantaged groups or impacts infrastructure that benefits all segments of society.

### Measures of child health

This paper examines three key measures of child health: neonatal mortality, child mortality, and height-for-age Z-score (HAZ). Each outcome is a well-established measure of both child health and the overall development of a particular country. Childhood deaths are often monitored by specific windows of time in a child’s life. For example, neonatal mortality refers to death in the first 28 days of life while child mortality is defined by deaths in the first 5 years of life. The neonatal period is the most critical time for a child and often represents deaths due to birth complications [53]. Child mortality includes the critical neonatal period in addition to other key developmental periods during the first 5 years. Recently, much progress had been made in reducing child mortality [54]. However, the neonatal period has not experienced similar reductions, resulting in neonatal mortality comprising an ever-increasing proportion of all childhood deaths [55–57].

Undernutrition is an important indicator of child health, remains common in low- and middle-income countries, and it contributes substantially to poor development in more than 200 million children worldwide [58], [59]. Stunting (low height-for-age) is often used to represent nutritional status in children and is a reflection of chronic undernutrition [60]. Determinants of undernutrition include food insecurity, chronic infections, low maternal education, inadequate breastfeeding, and poor socioeconomic conditions [59, 61]. Consequences of child undernutrition are profound as proper nutrition is critical to motor development, cognitive achievement, schooling, morbidity and mortality [59, 62–66].

The purpose of this study is to examine the extent to which several factors may mitigate socioeconomic inequality in key child health outcomes in selected sub-Saharan African countries. Specifically, the study will first explore the relationship between three measures of socioeconomic inequality and children’s nutritional status and mortality. Second, the study will consider the degree to which reproductive behavior, access to health care, and the broader macro context mitigate the relationship between socioeconomic measures and child health.

## Methods

The Demographic and Health Surveys (DHS) for Africa are the primary source of data for the analysis (http://www.measuredhs.com). Data collected since 2003 from 26 countries are analyzed to examine the impact of social determinants on child health. We focus on this time period because some of the measures in DHS are comparable for this period (the wealth index and a more detailed measure of maternal education.) Using this time period also allows for the assessment of change, as more attention has been given to social disparities in health outcomes. Several countries have multiple surveys. DHS surveys are co-sponsored by USAID, the governments of the countries where the surveys are conducted, and several other foundations. Surveys are based on national probability sampling so that results can be generalized to the country level. Trained interviewers visit selected households and conduct interviews with men and women of reproductive age. Interviewers also prepare a household roster with basic information for all members of the household. These surveys have become widely accepted sources of information for a variety of health related topics.

The key child health outcomes of interest are neonatal mortality (coded 0 or 1), the hazard rate of child survival until age five, and nutritional status as indicated by height-for-age Z-score multiplied by 100 to facilitate reporting of significant digits (HAZ). Measures of social status include maternal education treated as a interval level variable (no education, incomplete primary, complete primary, incomplete secondary, complete secondary, and post-secondary), wealth, a reflection of the household standard of living, as measured by household assets such as appliances and home building material sanitation facilities and housing construction, and urban/rural residency. Specific factors included in the wealth index vary from country to country (for details see http://www.dhsprogram.com/topics/wealth-index/Wealth-Index-Construction.cfm). Key moderating factors include prior birth interval (minimum of 24 months between births), presence of a skilled birth attendant (presence of doctor or nurse) at delivery, immunization (coded 1 if children received recommended immunizations including BCG, DPT 1 and Polio 1, and 0 otherwise) within 2 months of birth, year of the survey, per-capita income (GDP per capita), per capita expenditure on health and per capita expenditures on foreign aid in the 3 years prior to the survey. We only consider the first round of immunizations so we can include the youngest children in the analysis. The national level data on per capita income and per capita health expenditures were gathered from the World Bank [67]. If data for a specific DHS survey year were not available for a country, values within 3 years of the DHS survey year were used. Data on foreign aid were obtained from the AidData.org database [68]. Initially, we categorized aid by sectors including agriculture, health, reproductive health, water development, and all other aid. Per capita aid in all of these sectors except water were weakly associated with poor child health outcomes. Because we are interested in moderating factors that improve child health, analysis reported here only includes per capita foreign aid for water development.

**Fig. 1:**
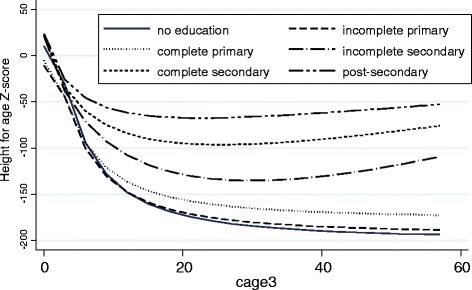
Educational inequality in children’s nutritional status (height/age z-score)

Several other household and child characteristics are associated with children’s health in developing countries [18]. This analysis includes maternal age, child’s age (in the models for nutritional status), child’s birth order, sex of the child, whether the child was a twin, presence of the father, marital status of the mother, household size, maternal employment and whether or not the father has at least some secondary education. Younger mothers may not be as likely to have resources and experience they can use to promote greater health for their children. As children age, their nutritional status (height-for-age Z-score) deteriorates relative to the WHO standard (see Fig. 1) because they do not receive adequate nutrition and are at risk of infections leading to diarrhea. Twins and children with more older siblings are at higher risk of mortality and undernutrition. Male children have higher rates of mortality but there is generally not a great gender difference in access to calories. The presence of a father in the home has been shown to be associated with better child outcomes [69–71]. For example, Dearden et al. found that children who saw their father daily or weekly at both one and 5 years of age had higher HAZ scores than children who saw their fathers less often at either or both ages (2012) [70]. Finally, father’s education provides an additional resource that may benefit children independent of maternal education and household wealth. We also include marital status of the mother, household size, and maternal employment to adjust for household structure and mother’s time availability. We considered including breastfeeding practices but measurement of this variable in DHS is not sufficient to capture the timing of exclusive breastfeeding and introduction of other foods into the diet.

### Analytical approach

Three regression models are used depending on the distribution of the measure of child health. Logistic regression is used to predict a dichotomous variable indicating mortality in the first month, Cox regression is used to predict child mortality measured in months, and linear regression is used for height for age z-scores. All countries and years are pooled. Regression models for neonatal mortality and nutritional status use multi-level models with country as the level two unit of analysis to account for intra-group correlations within countries. The Cox-regressions include fixed effects for each country. Stata 14.1 was used to estimate all models. Year of the survey, GDP per capita, health expenditures per capita and per capita aid for water development are measured at the national level. Forty-two percent of the households have more than one child under age 5. We estimated models adjusting standard errors for household clustering. Design effect statistics are all well below 2.0 (deff). Moreover, the standard errors in these models were only slightly larger and did not affect our conclusions.

Regression coefficients for the three social determinants, maternal education, wealth and urban residence, indicate the degree of socioeconomic inequality in health outcomes: larger coefficients show a steeper gradient of difference between more and less advantaged children. For example, a coefficient of 4.88 for maternal education implies that a child whose mother has post-secondary education will score .25 standard deviations higher on height-for-age than a child whose mother has no education ((4.88*5)/100 = .244), indicating substantial educational inequality. A coefficient of 2.0 would only imply a .10 standard deviation difference between children of the most and least educated mothers. Interaction terms between each of the moderating factors and the social determinants show the degree to which these factors have potential to reduce inequality. If coefficients for interaction terms run counter to the coefficients for social determinants then mitigation is implied. In other words, if the influence of social determinants becomes smaller as the magnitude of moderating variables increase then the main effect of the social determinant and the interaction effect will work in opposite directions. For example, if the coefficient for maternal education is 5.0 and the interaction between birth spacing (coded 0 for short interval and 1 for long interval) is -3.0 then the education gradient is 5.0 for children with a short birth interval and only 2.0 (5 + -3*1 = 2) for children with a longer birth interval, implying that a longer birth interval reduces educational inequality in child nutritional status.

## Results

Table 1 reports key measures of child health and socioeconomic status for each of the countries in the sample. There is substantial variation in each of these indicators. Nutritional status varies from nearly two standard deviations below the WHO reference in Burundi and Malawi to values less than one standard deviation below the reference in Ghana, Senegal and Egypt. A value below 2 is the standard measure of stunting. Neonatal mortality varies from below 20 deaths per 1000 births in Egypt to over 40 deaths per 1000 births in Guinea, Lesotho, Mali and Nigeria. The wealth measure is not reported in Table 1 because it is standardized in each country and has a mean value of zero. Education varies from values not far from 0 (meaning a majority of women have no education) to values above 3 indicating most women have at least some secondary education. The percent living in urban areas varies from below 10 % to nearly 50 %. In short, even though African nations tend to score lower on measures of development than other regions, there is still great variation in key measures of socioeconomic status.

**Table 1:** Means for nutritional status and socioeconomic variables

Country	Year	Sample size	Nutrition (HAZ)	Neonatal mortality	Education	Urban
Overall	--		-1.40	30.6	1.09	20.6
Burkina Faso	2003	10645	-1.50	28.7	.25	16.2
Benin	2006	16075	-1.54	31.2	.40	35.5
Burundi	2010	7742	-1.89	28.4	.88	17.6
Cameroon	2004	8125	-1.26	29.4	1.54	38.9
Egypt	2005	6661	-.81	19.9	2.30	36.4
	2008	10872	-.85	16.0	2.61	36.5
Ethiopia	2005	9861	-1.65	34.7	.42	13.8
	2011	11654	-1.44	35.6	.46	17.0
Ghana	2008	2992	-.91	32.0	1.53	33.4
Guinea	2005	6364	-1.30	41.2	.23	21.5
Kenya	2003	5949	-1.19	32.9	1.66	25.8
	2008	6079	-1.16	29.0	1.68	24.1
Liberia	2007	5799	-1.35	31.0	.93	35.1
	2009	4193	--	37.9	.88	39.0
Lesotho	2004	3697	-1.60	44.9	1.96	18.1
	2009	3999	-1.37	41.0	2.13	16.8
Morocco	2003	6180	-.70	26.9	2.63	43.4
Madagascar	2003	5415	-1.71	24.0	3.24	54.5
	2008	12448	-1.60	23.7	2.69	17.9
Mali	2006	14238	-1.29	44.4	.27	23.7
Malawi	2004	10914	-1.83	29.9	1.05	10.4
	2010	19967	-1.61	30.2	1.24	9.5
Mozambique	2003	10326	-1.66	35.9	.72	35.2
Nigeria	2003	6029	-1.47	49.1	1.40	35.1
	2008	28647	-1.41	39.1	1.58	26.6
	2010	5978	--	39.3	1.59	27.2
Niger	2006	9193	-1.79	29.0	.30	28.4
Namibia	2006	5168	-1.09	22.3	2.28	38.2
Rwanda	2005	8649	-1.73	36.8	1.05	19.7
	2007	5489	--	28.2	1.16	23.1
	2010	9002	-1.76	27.0	1.19	13.6
Senegal	2005	10944	-.79	33.2	.39	32.7
	2010	12326	-1.05	29.4	.43	29.6
Chad	2004	5635	-1.47	35.7	.44	44.4
Tanzania	2004	8564	-1.56	31.2	1.38	17.1
	2007	7502	--	25.7	1.46	16.4
	2010	8023	-1.45	28.2	1.46	18.8
Uganda	2006	8369	-1.37	26.4	1.18	11.0
	2009	4012	--	25.8	1.24	10.8
Zambia	2007	6401	-1.49	32.2	1.64	32.4
Zimbabwe	2005	5246	-1.24	23.8	2.22	25.5
	2010	5563	-1.20	26.6	2.55	29.0

Table 2 reports mean values of the moderating factors by country. A majority of births in each country are either first births or occur at least 24 months following the birth of a preceding child. Access to a skilled birth attendant (doctor or nurse) shows much greater variation from under twenty percent in Chad and Guinea to over seventy percent in Namibia. Although immunization rates were sub-optimal for full protection, the rates are generally high falling near or above ninety percent in several countries. But immunizations rates fall below seventy percent in a few cases. Per capita income varies widely across countries ranging from $220 to $2680. National expenditures on health care and foreign aid for water development also show substantial difference by national context. Not surprisingly, per capita income and per capita health expenditures are highly correlated (*r* = .95) such that results for these two variables will be similar. Correlations among social determinants (*r* < .6) and other moderating factors are much smaller (*r* < .25) so these other factors will have independent influences on health outcomes. Tests for multicollinearity indicate that with exception noted above, these data are not problematic (all VIFs < 2 and 1/VIF > .5).

**Table 2:** Means for moderating factors

Country	% prior birth interval > 24 months	% skilled birth attendant	% immunized (>2 months old)	Per capita income (1000 USD)	Per capita expenditure on health (100 USD)	Per capita foreign aid for water development (USD)
Burkina Faso	88.5	37.5	83.6	.28	.16	9.29
Benin	88.5	69.6	89.6	.52	.24	1.27
Burundi	82.8	63.4	98.3	.23	--	1.32
Cameroon	83.5	61.1	86.5	.89	.40	.28
Egypt	81.3	61.0	94.7	1.17	.66	1.94
Ethiopia	82.5	13.6	73.5	.22	.06	.66
Ghana	88.6	36.7	89.1	.53	.39	4.22
Guinea	88.6	19.3	77.9	.37	.19	.17
Kenya	80.4	43.5	92.7	.45	.24	1.55
Liberia	83.8	44.7	84.4	.30	.26	.88
Lesotho	92.5	57.2	94.1	.70	--	4.64
Morocco	81.2	42.7	90.1	1.19	.60	8.95
Madagascar	82.0	38.3	79.7	.39	.17	.19
Mali	81.9	22.7	76.0	.31	.21	.66
Malawi	87.7	62.5	93.8	.26	.18	1.45
Mozambique	88.4	30.1	85.2	.23	.12	.76
Nigeria	80.2	33.4	64.9	1.04	.66	.06
Niger	81.4	28.0	63.9	.26	.13	.18
Namibia	88.4	74.9	92.5	2.68	2.10	2.96
Rwanda	82.5	42.7	95.9	.35	.30	.83
Senegal	85.2	44.4	89.8	.80	.44	2.83
Chad	79.8	5.5	65.0	.33	.18	.59
Tanzania	86.4	42.2	93.4	.34	.18	1.49
Uganda	78.0	35.1	87.8	.34	.26	.87
Zambia	86.5	44.3	91.6	.50	.32	2.16
Zimbabwe	91.9	57.6	86.4	.58	--	.67

**Fig. 2:**
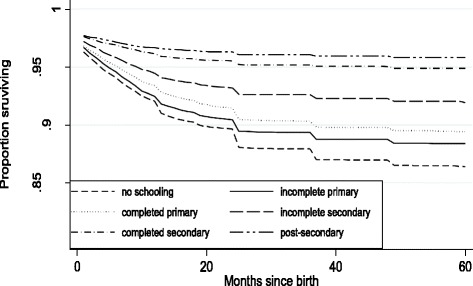
Educational inequality in child survival to age 5

Figures 1 and 2 illustrate the relationships between maternal education, nutritional status and child survival. Children whose mothers completed secondary school score three-fourths of a standard deviation higher on nutritional status than children whose mothers have no schooling. Children with more educated mothers also have significantly greater chances of surviving birth and early childhood. About 12 % of children whose mothers have no education do not live until their 5^th^ birthday compared to only about four percent if mothers completed secondary school. Differences by wealth and urban residence show similar patterns.

Multivariate regression is used to assess the independent effects of each variable (see Table 3) while taking into account other determinants of child health. In order to show more significant digits, height for age is reported in hundredths of a standard deviation. Consistent with prior research, each measure of status is associated with higher nutritional status (positive coefficients), lower neonatal mortality (coefficients below 1.0) and low rates of child mortality (coefficients below 1.0), with the exception that the wealth difference in neonatal mortality is small and statistically insignificant. In other words, there is substantial socioeconomic inequality in child health. Coefficients for control variables indicate that children have better health outcomes if the husband is not present, if the father has some secondary education, if there are fewer preceding siblings, if it is a singleton birth, if the child is female and if the mother is older, if the household is larger, and if the mother is married. It is possible that presence of the father detracts from resources available to children or that fathers who are absent because they are migrant workers contribute to household income. Larger household may have more people available to provide childcare and support. Results for maternal employment are mixed, but effects are not large.

**Table 3:** Baseline regression models predicting nutritional status, infant mortality and child mortality

	Nutritional status	Neonatal mortality (odds ratios)	Child mortality rate
Maternal education	4.88*	.956*	.914*
Urban residence	7.49*	.957*	.949*
Wealth	21.03*	.998	.942*
Control variables:			
Child age	-2.07*	--	
Husband present	-1.68	1.080*	1.078*
Husband-secondary education	8.69*	.957	.951*
Birth order	-3.45*	1.063*	1.081*
Twin	-32.13*	2.745*	1.993*
Female	14.78*	.717*	.859*
Maternal age	1.84*	.984*	.981*
Household size	.35*	.920*	.926*
Mother married	2.60*	.945	.879*
Mother employed	.10	1.086*	1.043*
Constant	-150.35	.099	--

*p < .05

The central question of this research is whether other factors moderate the impact of socioeconomic inequality. We address this question by including interaction terms between each potential moderating factor and each social determinant. Moderation is indicated if the coefficient for the interaction term has a coefficient that is opposite in sign to the main effect for the social determinant for nutritional status, and a coefficient that is greater than 1 for neonatal mortality and child survival. Tables 3, 4, 5, 6, 7 and 8 consider each of the factors we have included.

**Table 4:** Interaction of social determinants and presence of a skilled birth attendant

	Nutritional status	Neonatal mortality	Child mortality
Maternal education	5.14*	.971	.919*
Urban Residence	8.74*	.974	.977
Wealth	16.59*	1.007	.991
Skilled attendant	19.22*	.978	.874*
Skilled attendant*maternal education	-1.95*	.988	1.005
Skilled attendant*urban residence	-5.80*	.945	.947
Skilled attendant*wealth	4.38*	.986	.947*

*p < .05

Control variables included in model but not reported

**Table 5:** Interaction of social determinants and birth spacing

	Nutritional status	Neonatal mortality	Child mortality
Maternal education	6.21*	.906*	.878*
Urban Residence	11.42*	.937	.913*
Wealth	21.09*	.931*	.882*
Birth interval > 24 months	17.65*	.542*	.594*
Birth interval*maternal education	-1.72*	1.067*	1.052*
Birth interval*urban residence	-4.57	1.029	1.051
Birth interval*wealth	-.117	1.089*	1.084*

*p < .05

Control variables included in model but not reported

**Table 6:** Interaction of social determinants and immunization

	Nutritional status
Maternal education	7.63*
Urban Residence	3.48
Wealth	18.95*
Immunization	-14.14
Immunization* maternal education	-2.90*
Immunization *urban residence	4.86
Immunization *wealth	2.40

*p < .05

Control variables included in model but not reported

**Table 7:** Change in social determinants over time

	Nutritional status	Neonatal mortality	Child mortality
Maternal education	6.53*	.964*	.912*
Urban Residence	7.58*	.976	.938*
Wealth	21.74*	.938*	.912*
Year	2.72*	.974*	.941*
Year*maternal education	-.55*	.998	1.001
Year*urban residence	.08	.993	1.000
Year*wealth	-.28	1.017*	1.010*

*p < .05

Control variables included in model but not reported

**Table 8:** Change in social determinants associated with economic development

	Nutritional status	Neonatal mortality	Child mortality
Maternal education	7.39*	.954*	.906*
Urban Residence	11.75*	.915	.920*
Wealth	21.96*	1.052*	.980
Per capita Income (in 1000 USD)	12.24*	.771*	.582*
Per capita income*maternal education	-2.43*	1.011	1.017
Per capita income *urban residence	-5.20*	1.062	1.032
Per capita income*wealth	-1.57	.934*	.943*

*p < .05

Control variables included in model but not reported

**Fig. 3:**
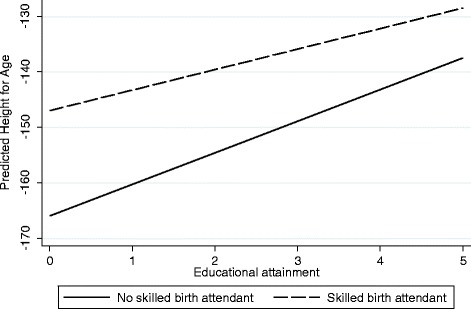
Relationship between Education and Nutritional Status by Presence of a Skilled Birth Attendant

Delivering with a skilled birth attendant (doctor or nurse) is associated with improved nutritional status (.19 standard deviation increase in height for age) and a 24 % reduction in child mortality, but has little impact on neonatal mortality (Table 4). Presence of a skilled birth attendant may more accurately reflect broader access to care rather than the care provided at the delivery of the child. Access to health care, as indicated by having a skilled birth attendant at delivery also reduces the impacts of maternal education and urban residence on nutritional status, but not on child survival. In contrast, access to health care increases the impact of wealth on child outcomes suggesting the wealthy gain more advantage from access to health care than do the poor. The graph in Fig. 3 illustrates this pattern. Predicted values of height-for-age increase with maternal education and are higher for children delivered by a skilled birth attendant. But the relationship between maternal education and nutritional status is less pronounced among children delivered by a skilled birth attendant. In other words, access to health care reduces the inequality in nutritional status associated with maternal education. Results for birth spacing (Table 5) indicate that births occurring at least 24 months after the preceding birth result in better nutritional status (.17 standard deviations higher), a 45 % reduction in neonatal mortality rates and a 35 % reduction in child mortality. Moreover, the influence of maternal education is smaller when the preceding interval is at least 24 months. Interactions for urban residence are not statistically significant. Interactions between wealth and birth interval are significant in two of the three models and in the expected direction. In short, maternal education and wealth do not have as large an impact on child health when births are spaced at least 24 months apart. Immunization has a negligible relationship with child nutritional status (Table 6). This may be due to the overall high rates of immunization and the fact that immunizations do not have a large direct impact on nutritional status. Interactions between immunization and education, and immunization and urban residence are small and statistically non-significant. In contrast, the interaction between immunization and wealth does have a positive coefficient. As with access to a skilled birth attendant, this finding suggests that the wealthy benefit more from access to health care.

Each indicator of child health has improved over time, but the gains have been small (Table 7). Moreover, the gains are associated with only modest declines in some aspects of inequality. The impact of maternal education on nutrition and child survival is diminishing, as is the impact of wealth on neonatal mortality and child survival. Urban/rural differences in child survival are also declining. Other interaction terms are not statistically significant and most of the interaction terms are small suggesting that the changes observed between 2003 and 2011 are making a modest dent in inequality at best.

As countries experience economic development, nutritional status improves (.11 standard deviations per $1000 in per capita income) and mortality rates decline. Moreover, some health inequalities decline with development (Table 8). Specifically, as per capita income increases, nutritional status improves more among the uneducated, rural residents and the poor in comparison with more advantaged groups. Educational inequality in child survival also declines. But the impact of wealth on neonatal mortality actually increases with economic development. In short, economic development may provide a partial, but incomplete avenue for reducing health disparities.

Countries that spend more per capita on health have better child nutrition and lower mortality rates (Table 9). But increasing expenditures only moderates two of the observed inequalities, namely urban/rural differences in nutritional status and educational differences in child survival. Wealth differentials in neonatal mortality actually increase with rising expenditures, suggesting that the wealthy benefit more than the poor from general improvements in the health care system. Thus, the investments these countries have made in health care have not been particularly effective in reducing health disparities.

**Table 9:** Change in social determinants associated with per capita health expenditures

	Nutritional status	Neonatal mortality	Child mortality
Maternal education	5.28*	.945*	.902*
Urban Residence	12.56*	.910	.903*
Wealth	21.98*	1.050*	.973
Per capita health expenditures (in 100 USD)	24.00*	.679*	.401*
Per capita expenditures*maternal education	-1.16	1.030	1.029
Per capita expenditures *urban residence	-10.19*	1.107	1.073
Per capita expenditures*wealth	-1.09	.886*	.928*

*p < .05

Control variables included in model but not reported

Finally, we consider foreign aid (Table 10). Foreign aid for water development is associated with modest gains in child nutritional status and lower neonatal mortality. Aid to improve water also has a modest impact on reducing some aspects of inequality, but the patterns are very inconclusive. Interactions suggest that this type of aid reduces both educational and urban/rural disparities in nutritional status but increased wealth inequality in child nutrition. But coefficients for child survival suggest just the opposite, and coefficients for neonatal mortality are small and statistically non-significant. In short, no clear pattern is evident.

**Table 10:** Change in social determinants associated with per capita aid for water development

	Nutritional status	Neonatal mortality	Child mortality
Maternal education	5.32*	.949*	.910*
Urban Residence	9.02*	.955	.947*
Wealth	19.44*	1.010	.944*
Per capita water aid	1.48*	1.001	.990*
Per capita water aid *maternal education	-.41*	1.006	1.003
Per capita water aid *urban residence	-.73	.997	.993
Per capita water aid *wealth	.77*	.994	1.001

*p < .05

Control variables included in model but not reported

## Discussion

Socioeconomic inequality is a defining characteristic of children’s health, even in countries with low levels of economic development, pervasive undernutrition and high rates of infant mortality. This analysis documents large inequalities based on mother’s education and household wealth in Africa. Once these are taken into account the effect of urban/rural residence is smaller, but not trivial.

Wealth, as measured by assets and availability of services, is often used as an indicator of economic position in developing countries because income is unstable and because subsistence agriculture and many exchanges do not rely on cash. The most obvious reason for a relationship between economic position and children’s health and nutrition is that access to health care and nutritious food cost money that the poor cannot afford. Further, other material deprivation resulting from poverty such as dirty water, lack of sanitation, and poor housing also contributes to poor child health outcomes [1]. Thus, household wealth often has a profound impact on children’s health in developing countries [71] and on health seeking behavior, especially in the modern health sector [72]. Our results indicate that children in wealthier households do have better nutritional status and lower mortality rates. Wealth does not have a statistically significant relationship with neonatal mortality, suggesting that the health risks during delivery and the first few weeks of life are not reduced in households with more economic resources.

Children also have improved nutritional status and lower mortality if their mothers are more educated. Possible pathways linking maternal education and child health include access to health care, health knowledge and good health practices. Frost and colleagues (2005) found that maternal education influenced child nutritional status primarily through the pathways of socioeconomic status and modern attitudes regarding health care [17]. Additionally, maternal schooling has also been found to be a key predictor of whether children in low- and middle-income countries experience growth recovery or growth faltering [73]. Education may also increase cognitive ability [74], human and cultural capital [75] and use of modern health services [15–17, 76, 77]. Educated mothers are better able to access, understand, and respond to health information designed to improve child health in resource poor settings resulting in maternal education receiving special attention in relation to child health outcomes. For example, research suggests that schooling enables mothers to make more informed decisions about nutrition, hygiene and preventative care ([15, 16]. Further, research in Northern Kenya found that even in areas that lack formal education, maternal health knowledge, regardless of education, results in reduced infant illness [78]. Studies from Cambodia and Bolivia found that much of maternal education’s effect operates through socioeconomic indicators such as occupation type, household wealth and type of earnings, and that more educated women tend to have more educated husbands [17, 79]. Lastly, educated mothers are not only more likely to obtain secure employment, they are also more likely to utilize healthcare and engage in behaviors that improve child nutrition [80, 81].

Our results confirm that children in rural areas are at greater risk of death and undernutrition [19]. Only the most severely disadvantaged urban children have health outcomes on par with their rural counterparts [82–84]. The rural-urban disparity in child health is found in nations of various developmental levels throughout the developing world [14, 84–90]. More developed infrastructure and public services in urban areas directly affect the health resources available to residents [15]. In contrast, limited economic and educational opportunities in rural areas have significant implications for residential disparity in child health outcomes [16, 90]. Rural areas are also slower to adopt contraception and often develop community-level values of marriage and fertility that reinforce reproductive ideals and norms [91, 92].

## Conclusions

The primary goal of this research is to identify means of reducing these inequalities. The most common result we find is persistent inequality—35 of the 57 tests for interactions are not statistically significant. But 17 of the tests suggest that inequality can be reduced. Although results are not parallel across each of the three outcomes considered here, they do suggest potential progress has been or can be made to reduce the disadvantage of being born to a mother with little or no education. Over time the educational disparities have declined to a degree. Longer birth spacing, utilization of skilled birth attendants, economic development, and foreign aid to promote water development are especially beneficial to the children of educationally disadvantaged mothers. Results for the urban/rural gap are less promising. However, the urban/rural gap is not as large as the education gap. Having a skilled birth attendant, economic development and expenditures for health reduce some of the urban/rural gap in child health, but each of the other mechanisms have little bearing the urban/rural gap.

Results for wealth paint a different picture. Longer birth spacing does reduce the wealth disadvantage to some degree. As might be expected, results imply that the wealthy benefit more from the services of a skilled birth attendant, from higher per capita expenditure on health, and from aid to improve water. This does not mean that provision of health services is a bad policy since each socioeconomic group benefits. Rather the socioeconomically advantaged may benefit more from these services. The implication is that programs and policies aimed at increasing access to health services need to focus these efforts toward the poorest households.

Our study has several limitations. As with any cross-sectional analysis, causal influence can only be inferred from observed relationships. We have only considered some of the most relevant socioeconomic inequalities and mechanisms that may mitigate inequality. Moreover, our sample is limited to the African countries that have opted to participate in the DHS program. We only have national level data. There is substantial variation in social conditions and health within countries. Aid and health projects are often targeted to specific areas rather than to countries as a whole. Unfortunately, data on per capita and health expenditures are only reported at the national level.

Another disadvantage of our approach is that we cannot adequately distinguish the independent influence of each of the interactions. While per capita income and per capita health expenditure are highly correlated, the correlations among measures of social inequality and moderating variables are not particularly high and indicators of multicollinearity are small. However, when the interaction terms are added coefficients become unstable and multicollinearity indicators become inflated. Thus, our conclusions should not be interpreted as strong recommendations regarding specific moderating forces but as pointers to the types of moderators that should be considered. Lastly, further research examining other social determinants could provide additional insights to our findings. For example, ethnicity, religion, maternal age, sex of child, and social capital potentially impact children’s health and may mitigate or facilitate inequalities.

Only data at the national level within country would be good.

Vaccinations limited to 2 months in order to include more children—full impact important.

Although this study does not include an exhaustive list of social determinants, mechanisms and broader social trends, results suggest there is no silver bullet that will eliminate the socioeconomic disadvantages that heavily influence child health in the developing world. Efforts to reduce health disparities may not be effective because of lack of coverage, lack of quality, and the lack of sustainability of health services [44]. Measures for birth spacing and immunization have values near the maximum, but this does not imply that they should not be targets for further health policy. Spacing beyond the arbitrary cutoff of 24 months used here does benefit children, and we have included reports on only three vaccinations. The greatest challenge to reducing health disparities may occur because the most economically advantaged are more likely to benefit from economic development and improvements in health care services. Given the substantial health disparities observed in Africa, one logical approach to improve overall health would be to focus on the most disadvantaged groups who are at highest risk of undernutrition and death. Although our findings suggest health promotion can provide great benefit to the most disadvantaged groups as is the case with birth spacing, it is also likely that current implementation of these programs may benefit the rich even more than the poor as was shown by results examining the provision of a skilled birth attendant. Our results imply that significant reductions in health disparities will not necessarily occur unless this becomes an explicit goal of health policy.
